# Using GIS to Identify Priority Sites for Colorectal Cancer Screening Programs in Texas Health Centers

**DOI:** 10.5888/pcd20.220205

**Published:** 2023-03-02

**Authors:** F. Benjamin Zhan, Yanyan Liu, Mei Yang, Nicole Kluz, Todd A. Olmstead, Jenny Spencer, Navkiran K. Shokar, Roxana L. Cruz, Michael P. Pignone

**Affiliations:** 1 Texas Center for Geographic Information Science, Department of Geography and Environmental Studies, Texas State University, San Marcos, Texas; 2 Cancer Prevention and Control Program, LiveStrong Cancer Institutes, Dell Medical School, University of Texas at Austin; 3 Department of Population Health, Dell Medical School, University of Texas at Austin; 4 Department of Internal Medicine, Dell Medical School, University of Texas at Austin; 5 University of Texas at Austin, LBJ School of Public Affairs, Austin, Texas; 6 Texas Association of Community Health Centers, Austin, Texas

**Figure:**
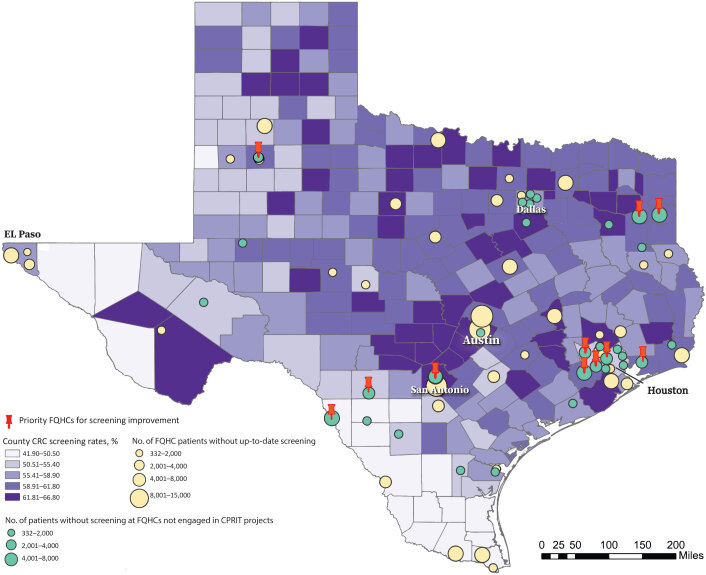
The map shows priority FQHCs with the highest number of age-eligible patients (aged 50–74 y) without up-to-date screening in December 2020 and without CPRIT funding as of December 2021 (Roxana L. Cruz, PhD, personal communication, July 6, 2022), screening rates at the county level in 2018 (1), and FQHCs in Texas, including those that had not engaged in CPRIT projects as of December 2021. Circles are the county locations of administrative offices of the FQHCs, and sizes of circles represent the number of patients without up-to-date screening at the FQHCs. Yellow circles indicate FQHC patients without up-to-date screening and engaged in CPRIT projects, and green circles indicate FQHC patients without up-to-date screening at FQHCs not engaged in CPRIT projects. The total number of these patients in the 11 FQHC service areas was 40,163; the number of patients in each of the 11 FQHCs ranged from 2,086 to 5,383. Abbreviations: CPRIT, Cancer Prevention and Research Institute of Texas; CRC, colorectal cancer; FQHC, federally qualified health center.

## Background

Colorectal cancer (CRC) screening is effective in reducing CRC incidence and death, but it is underutilized, particularly in under-resourced communities and populations (2–11). Federally qualified health centers (FQHCs) serve populations at high risk for being unscreened, including uninsured and rural populations, making them an important focus for efforts to improve CRC screening and reduce screening inequities (7–11). These efforts are challenging, particularly in states like Texas where 40% of patients seen in FQHCs are uninsured; the proportion of patients up to date with CRC screening in the 72 FQHCs in Texas was only 34.2% in 2020 and 36.2% in 2021 (1). Texas fell below the national FQHC screening rate of 42% in 2021 (12), the overall national screening rate of 65.2% from 2018, and the Healthy People 2030 national goal of 74.4% (13). Improving the use of preventive care like colorectal cancer screening is a strong priority for FQHC systems and the federal government.

The Cancer Prevention and Research Institute of Texas (CPRIT) funds CRC screening programs throughout Texas, with a focus on disproportionately affected populations, but its reach is incomplete (14). The vast area of the state creates geographic challenges, including lack of access to primary and specialty medical services. To increase CRC screening rates among age-eligible FQHC patients in Texas through the implementation of evidence-based CRC screening programs such as mailed fecal immunochemical testing kits (9–11), it is necessary to identify FQHC systems that may benefit from outreach and assistance in growing or building new programs.

We obtained data from multiple sources, conducted geospatial analysis, and identified and mapped a list of priority FQHCs in Texas where the number of FQHC patients without up-to-date CRC screening is high and participation of the FQHCs in CPRIT-funded CRC screening projects is absent. In the next phase of the research, we will use the mapped information to contact and consult with the FQHCs and help them apply for CPRIT funding to enhance CRC screening in their systems. We sought to use data from multiple sources to conduct geospatial analysis with the goal of identifying and mapping a group of priority FQHC systems in Texas for outreach and consultation on efforts to increase CRC screening.

## Data and Methods

We collected data from the Centers for Disease Control and Prevention (CDC), the US Census Bureau, the Uniform Data System eMapper, the Texas Department of State Health Services (Texas DSHS), and the Texas Association of Community Health Centers (TACHC). CDC data included the estimated 2018 CRC screening rates for people aged 50 to 74 years at the county and the Zip Code Tabulation Area (ZCTA) levels (Roxana L. Cruz, PhD, personal communication, July 6, 2022). Census data included population counts for people aged 50 to 74 at the county and ZCTA levels in 2020. Data from the Texas DSHS included boundaries of health service regions and trauma service areas in the state.

Data from TACHC included the 2020 CRC screening rates among patients served by each of the 72 FQHCs in Texas and information about whether an FQHC had participated in any CPRIT-funded project as of December 2020, according to both CPRIT and TACHC. Geographic data about the FQHCs included the location of the administrative office and service area of each FQHC. The service area of an FQHC includes different ZCTAs across county boundaries, and patients living in the same ZCTA may be covered by different FQHCs. Because the service areas of different FQHCs may overlap, we used the locations of the FQHC system’s administrative offices when mapping the information associated with each FQHC. Because the boundaries of the service areas of the FQHCs, ZCTAs, and counties do not necessarily line up well, analyses associated with these area units were performed separately.

We completed several geospatial analyses to identify priority FQHC systems for CRC screening outreach in Texas in 2022. First, we estimated the population aged 50 to 74 without up-to-date CRC screening in each county and ZCTA in Texas in 2020 based on the 2018 CRC screening rates and the population counts of people aged 50 to 74 years in 2020 at both the county and ZCTA levels. Second, we produced lists of counties and ZCTAs with the highest number of age-eligible people without up-to-date screening. These lists provide information about the priority areas at the county and ZCTA levels for CRC screening improvement in the general population.

Third, we used data from the 72 FQHC systems in Texas to calculate the number of age-eligible patients (50 to 74 y) without up-to-date screening in December of 2020. The data from TACHC contained data about the number of patients aged 50 to 74 seen at each FQHC during calendar year 2020 and the CRC screening rates of the patients seen at each FQHC. Fourth, we generated a list of priority FQHCs. Each FQHC on the list had at least 2,000 age-eligible patients without up-to-date screening in December 2020 and had not participated in any CPRIT-funded projects as of December 2020. The number 2,000 was selected because it was considered the minimum size necessary to justify a CPRIT program. We used ArcGIS Pro version 2.9.3 (Esri) to build the geodatabases, perform the analyses, and generate the maps. In this article, we discuss FQHC screening rates only; results at the ZCTA level are not presented. 

## Highlights

The geospatial analysis produced a list of 11 FQHCs (without current CPRIT funding) where improvements in CRC screening among age-eligible patients could have the largest impact (Table). The identified FQHCs are scattered throughout Texas and mainly located in east Texas, southwest Texas toward the Texas–Mexico border, and in the Texas Panhandle area. Mapping these FQHCs gives policy makers, researchers, and practitioners the needed information to develop and implement more effective CRC screening programs aimed at improving CRC screening uptake in communities that are underserved due to limited access to medical services, including patients served by FQHCs. The map helps readers clearly see the geographic distribution of the FQHCs where additional support could have the highest impact and demonstrates that the analytical power and mapping capabilities of geographic information systems can enhance CRC screening and other cancer prevention programs.

**Table:** Priority Federally Qualified Health Centers for Colorectal Cancer Screening Improvement (N = 11), Texas, United States, 2022

Health center	County^a^	No. of patients aged 50–74 y	CRC screening rate, %	No. of patients aged 50–74 y without up-to-date screening
A	Harrison	8,120	33.7	5,383
B	Bexar	8,316	35.9	5,326
C	Maverick	8,510	39.2	5,175
D	Fort Bend	5,356	18.0	4,392
E	Gregg	4,446	5.6	4,197
F	Chambers	3,177	0.9	3,148
G	Harris	4,111	23.6	3,142
H	Lubbock	3,650	20.8	2,889
I	Harris	2,341	2.5	2,283
J	Harris	2,536	15.5	2,142
K	Uvalde	3,091	32.5	2,086

Abbreviation: CRC, colorectal cancer.

a County in which the administrative office of an FQHC system is located. The number of patients and screening rate presented are associated with the FQHC system, not a county.

## Action

Members of the research team will use the mapped information to approach the FQHCs on the priority list, offer consultation to leaders at these FQHCs, form partnerships with these FQHCs to improve CRC screening, and help a subset with the strongest interest to apply for program funding from CPRIT or other sources. Ultimately, we hope the mapped information will help focus efforts to increase CRC screening rates for patients served by FQHCs in Texas and help reduce the prevalence of CRC in the state.
